# Prize-Giving at Charing Cross Hospital Medical School

**Published:** 1904-12-03

**Authors:** 


### PRIZE-GIVING AT CHARING CROSS HOSPITAL MEDICAL SCHOOL.

The annual presentation of scholarships, medals, prizes, and certificates to successful students of the Medical School attached to Charing Cross Hospital, took place on Wednesday, the 23rd inst., Sir Squire Bancroft in the chair.

The Dean, Mr. F. H. Waterhouse, in presenting his report, congratulated the school on the fact that the buildings were at length completed. Bedford Court, formerly an eyesore, had been acquired, and a new biological laboratory, curator's room, and physical laboratory erected on the site. Alluding to the improvements in the hospital, Mr. Waterhouse said the new surgical block was completed with the exception of the operating theatres, which awaited furnishing and fittings, and it was hoped that it might be opened early in 1905. During the two months in which the hospital was closed, the students were received at King's College, the Middlesex, and Westminster Hospitals. An important innovation had been introduced in the opening of the summer session 10 days earlier than formerly, in order to avoid interference with the work occasioned by the July examinations. A new prize had been founded through the generosity of Dr. T. Henry Green, who after resigning from the active staff of the hospital, had been appointed Emeritus lecturer on clinical medicine. He had devoted the whole amount of the £200 subscribed as a testimonial to the endowment of this prize. He recorded with regret the death of Dr. Alfred Sangster, consulting physician since 1894. Dr. Mitchell Bruce, who might be called one of the founders of the modern Charing Cross School, had resigned after 30 years' able and successful work, and had been appointed consulting physician to the hospital. The success of the Charing Cross students in the various examinations during the year had been up to its usual high average; there was an increase in the number of medical students as compared with last year, 75 having joined during 1904, but the number of dental students showed a slight decrease. The number of students—227—was likely to be largely increased in the near future, when the new hospital buildings, with a larger number of beds available for clinical teaching were completed, and it was anticipated that the removal of King's College Hospital to South London might also affect the numbers of those entering for training.

Sir Squire Bancroft, in presenting the prizes, after making a humorous allusion to his inability to compete with Sir Charles Wyndham, in throwing open his theatre to the staff and students, exhorted the unsuccessful not to be discouraged by their failure in examinations. This in itself was no discredit, and he trusted no one present would be disheartened in the work of life by so small a matter. Having paid a warm tribute to the kindly relations existing between the medical and theatrical professions, Sir Squire Bancroft said actors would always feel grateful to his Royal Highness the Prince of Wales for the words uttered by him at the last meeting of the Council of King Edward's Hospital Fund, in which he acknowledged what he characterised as the frequent, substantial, and unassuming help received by the London hospitals from the theatrical profession. Reminding his hearers that youth and hope were their stock-in-trade for the business of life, the speaker said for his own part he believed that opportunity knocked once at every man's house and he exhorted them to be ready for it when it came. As a layman he ventured to urge them to be cheerful, especially in the sick-room, and not to assume, in the presence of the patient, the mien of the undertaker. He concluded by cordially wishing them success in the career they had chosen—a career in which there was ample scope for those noble attributes, courage and self-sacrifice, as instanced in the lives of medical men who had enriched the world no less by their example than by their contributions to the sum of human knowledge, reminding them that laurels and renown were open to them also.

Mr. Leigh Wood, speaking on behalf of the Council, referred to the successful efforts of Sir Squire Bancroft on behalf of charities, resulting in the aggregate to considerably more than £15,000, and an expression of warm appreciation of his kindness on that and many other occasions, concluded the ceremony.

